# Supplementary material for: Asynchronous magnetic resonance elastography: Shear wave speed reconstruction using noise correlation of incoherent waves
Source: Magn Reson Med. 2022 Oct 27;89(3):990–1001. doi: 10.1002/mrm.29502 (PMC9792433; doi:10.1002/mrm.29502)
Supplement: Supplementary file 1 — DATA S1 MATLAB code used to generate the numerical simulations of Figure 1 [file MRM-89-990-s002.zip › k-Wave/helpfiles/example_tvsp_transducer_field_patterns.html]

Simulating Transducer Field Patterns Example (k-Wave)


# Simulating Transducer Field Patterns Example

This example demonstrates the use of k-Wave to compute the field pattern generated by a curved single element transducer in two dimensions. It builds on the Monopole Point Source In A Homogeneous Propagation Medium, Recording The Particle Velocity, and Defining A Sensor Mask By Opposing Corners Examples.

- Open the file in the MATLAB Editor
- Run the file in MATLAB

## Contents

- Defining the time varying pressure source
- Recording the statistics of the pressure field
- Running the simulation

## Defining the time varying pressure source

As in the previous examples, a time varying pressure source is defined by assigning a binary source mask to `source.p_mask` (which defines the position of the source points) along with a time varying source input to `source.p`. Here a single sinusoidal time series is used to drive a curved transducer element.

```
% define a curved transducer element
arc_pos = [20, 20];         % [grid points]  
radius = 60;                % [grid points]
diameter = 81;              % [grid points]
focus_pos = [Nx/2, Nx/2];   % [grid points]
source.p_mask = makeArc([Nx, Ny], arc_pos, radius, diameter, focus_pos);

% define a time varying sinusoidal source
source_freq = 0.25e6;       % [Hz]
source_mag = 0.5;           % [Pa]
source.p = source_mag * sin(2 * pi * source_freq * kgrid.t_array);

% filter the source to remove any high frequencies not supported by the grid
source.p = filterTimeSeries(kgrid, medium, source.p);
```

## Recording the statistics of the pressure field

To visualise the acoustic beam produced by the curved transducer, a sensor mask covering the entire computational domain is defined using the grid coordinates of two opposing corners of a rectangle (a list of the different sensor mask types is given in the Homogeneous Propagation Medium Example). If only the total beam pattern is required (rather than the beam pattern at particular frequencies or times), this can be produced without having to store the complete time series at each sensor point by setting `sensor.record` to  `{'p_final', 'p_max', 'p_rms'}`. With this option, at each time step k-Wave only updates the maximum and rms values of the pressure at each sensor point. This can significantly reduce the memory requirements for storing the sensor data, particularly if sensor masks with large numbers of sensor points are used.

```
% create a sensor mask covering the entire computational domain using the
% opposing corners of a rectangle
sensor.mask = [1, 1, Nx, Ny].';

% set the record mode to capture the final wave-field and the statistics at
% each sensor point
sensor.record = {'p_final', 'p_max', 'p_rms'};
```

Note, if recording the maximum or minimum pressure everywhere, it is also possible to set `sensor.record` to  `{'p_max_all', 'p_min_all'}`. This returns the maximum and minimum pressure everywhere, regardless of the shape or type of sensor mask. Consequently, provided no other outputs are required, in this case it is possible to leave the sensor mask blank by setting `sensor.mask = []`.

## Running the simulation

The simulation is again invoked by calling `kspaceFirstOrder2D`. To allow visualisation of the source elements within the grid, the source mask is assigned to the optional input `'DisplayMask'`. This mask is overlaid onto the plot during the simulation. The PML is also set to be outside the computational grid defined by the user and then hidden from display by setting the optional inputs `'PlotPML'` and `'PMLInside'` to `false`.

```
% create a display mask to display the transducer
display_mask = source.p_mask;

% assign the input options
input_args = {'DisplayMask', display_mask, 'PMLInside', false, 'PlotPML', false};

% run the simulation
sensor_data = kspaceFirstOrder2D(kgrid, medium, source, sensor, input_args{:});
```

As described in the Recording The Particle Velocity Example, if an input for `sensor.record` is defined, the output `sensor_data` returned from the simulation is defined as a structure, with the recorded acoustic variables appended as structure fields. In this case the output `sensor_data` is given as a structure with the fields `sensor_data.p_final`, `sensor_data.p_max`, and `sensor_data.p_rms`. For a sensor mask defined using the grid coordinates of opposing corners of a rectangle, `p_rms` and `p_max` are indexed as `(x_index, y_index)`, where `x_index` and `y_index` correspond to the grid index within the rectangle. The final pressure field along with the maximum and rms beam patterns are plotted below. Both the transducer focus and the side lobes are clearly visible.
